# EGFR-Mutant Urothelial Carcinoma Harboring an Ala750_Ile759delinsGlyGly Alteration with a Primary Resistance to Polychemotherapy and a Sensitivity to Osimertinib: A Literature Review on EGFR Alterations and Response to EGFR Tyrosine Kinase Inhibitors in Cancers

**DOI:** 10.3390/jcm14093129

**Published:** 2025-04-30

**Authors:** Jean-Baptiste Barbe-Richaud, Antonin Fattori, Véronique Lindner, Caroline Schuster, Gabriel Malouf, Erwan Pencreach, Laura Somme

**Affiliations:** 1 Oncology Department, Institut de Cancérologie Strasbourg Europe, 17 Rue Albert Calmette, 67200 Strasbourg, France; jb.barbe-richaud@icans.eu (J.-B.B.-R.); c.schuster@icans.eu (C.S.); g.malouf@icans.eu (G.M.); 2 Pathological Department, Hôpital de Hautepierre, 1 Avenue Molière, 67200 Strasbourg, France; antonin.fattori@chru-strasbourg.fr (A.F.); veronique.lindner@chru-strasbourg.fr (V.L.); 3 Molecular Departement, Hôpital de Hautepierre, 1 Avenue Molière, 67200 Strasbourg, France; erwan.pencreach@chru-strasbourg.fr

**Keywords:** deletion exon 19, *EGFR*, uncommon mutation, urothelial carcinoma, osimertinibkey points

## Abstract

Urothelial carcinoma is three to four times more common in men than in women, with a 73-year old mean age at diagnosis which is older than the average age at diagnosis of all cancers. Urothelial carcinoma is rare in people under 40 years of age. Smoking, exposure to industrial chemicals, and family history influence the development of bladder cancer, but age remains one of the most important risk factors. It is well established that women are more likely to be diagnosed with an advanced disease, impacting the prognosis and a higher stage-for-stage mortality compared to men. A gender difference is also observed when considering molecular features; for example, there a higher male/female ratio in *Fibroblast Growth Factor Receptor 3 (FGFR3)*-mutated bladder cancer. *Epidermal Growth Factor Receptor (EGFR)* amplifications, which are roughly depicted in 25–50% of urothelial carcinoma, have been correlated with a worse prognosis. Genomic alterations of clinical interest are mainly *Human Epidermal Growth Factor Receptor 2* mutations and amplifications, as well as *FGFR 3* alterations; however, no *EGFR* mutation has been routinely reported despite the frequency of its amplifications. Recurrently, no targeted inhibitors have demonstrated a benefit compared to platinum-based chemotherapy. We report a rare case of a 35-year-old woman presenting bone, hepatic, and lymph node metastatic urothelial carcinoma, harboring a deletion of 24 nucleotides in exon 19 of the *EGFR* gene with a 5-month response to osimertinib, a third-generation EGFR tyrosine kinase inhibitor.

## 1. Introduction

Urothelial carcinoma typically affecting older men with risk factors such as tobacco exposure. *Epidermal Growth Factor Receptor (EGFR)* amplifications have been identified in a significant proportion of urothelial tumors, yet EGFR mutations remain exceedingly rare and their clinical significance poorly understood. While EGFR tyrosine kinase inhibitors (TKIs) have transformed the management of EGFR-mutant non-small cell lung cancer, their role in other malignancies remains under investigation. Here, we report a rare case of a young female patient with metastatic urothelial carcinoma harboring an atypical EGFR exon 19 deletion (Ala750_Ile759delinsGlyGly), who demonstrated a primary resistance to platinum-based chemotherapy but experienced a clinically significant response to osimertinib. This case highlights the potential relevance of comprehensive genomic profiling in atypical clinical scenarios to personalize the therapeutic management. 

## 2. Case Presentation

- A urothelial carcinoma diagnosis in a young woman without risk factors.
- An uncommon deletion in exon 19 of the *EGFR* gene in metastatic urothelial carcinoma.
- Sensitivity to osimertinib despite the lack of efficacy with platinum-based chemotherapy.
- Importance of molecular assessment in young patients with no risk factors to identify targetable oncogenic drivers.
- Efficacy of EGFR tyrosine kinase inhibitors in *EGFR*-mutant cancers, regardless of histology.

**Figure 1:**
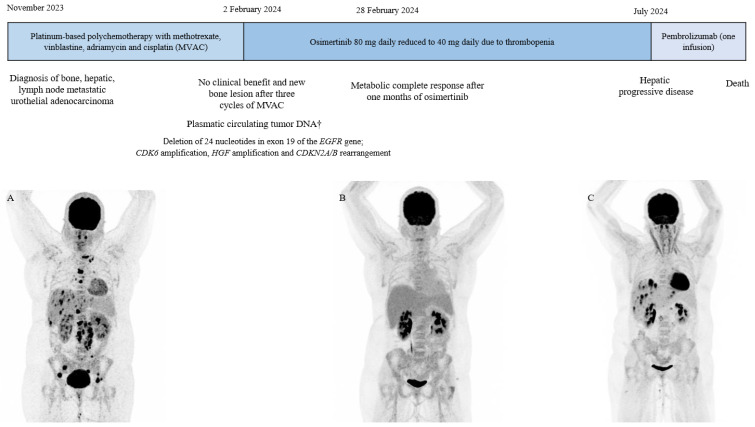
Timeline of diagnostic and therapeutic management; ^†^ liquid biopsy Fondation One test, MIP maximum intensity projection of 18^F^-FDG PET/CT: (**A**) multi-metastatic urothelial carcinoma at diagnosis, (**B**) metabolic complete response after one month of osimertinib and (**C**) hepatic progression after five months of osimertinib, suggesting an acquired resistance to osimertinib.

**Figure 2:**
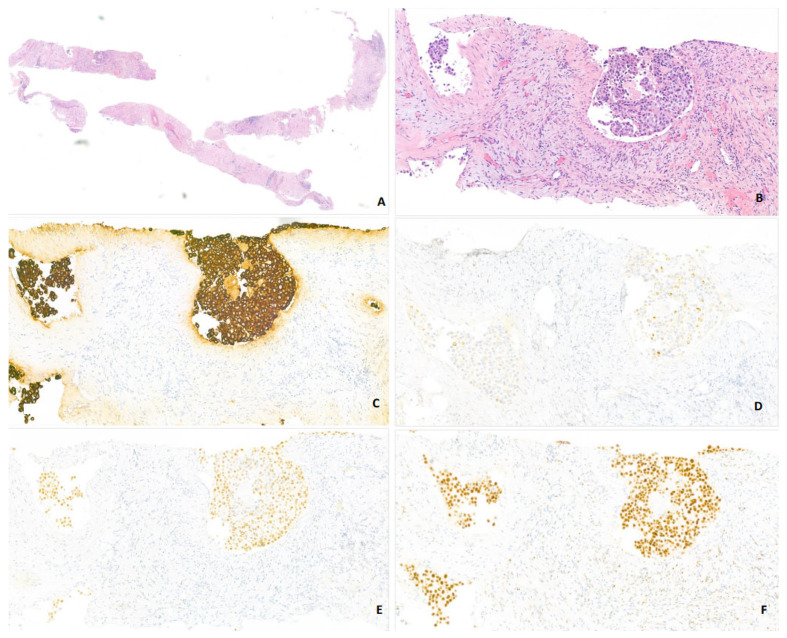
Biopsy sample of a left retroperitoneal lymph mass: Three cores measuring between 0.5 and 1.2 cm in length, composed of fibrous tissue containing a few vascular structures. The lumina of these vessels are almost entirely filled by a neoplastic proliferation of medium to large cells, characterized by large, round to oval, nucleolated nuclei arranged in cohesive clusters ((**A**,**B**): H&E, ×3 and ×25). On immunohistochemistry, these carcinoma cells were stongly and diffusely positive for Keratin 7 (clone OV-TL 12/30) ((**C**): ×25) and showed more focal and weak positivity for Keratin 20 (clone SP33) ((**D**): ×25). Strong and diffuse positivity was observed for P40 (polyclonal) ((**E**): ×25) and GATA3 (clone L50-823) ((**F**): ×25), while complete negativity was noted for TTF-1 (clone 8G7G3/1) and Estrogen receptor (clone SP1) (not shown).

In November 2023, a 35-year-old woman with no significant medical history presented with debilitating lumbar pain. A thoraco-abdominal CT scan revealed an enlarged left kidney (13.2 cm) with heterogeneous enhancement of the upper two-thirds, a thrombus of the left renal vein associated with multiple retroperitoneal lymph nodes, particularly in the left para-aortic region at the level of the left renal pedicle, hepatic metastasis, and a left corpus luteum lesion. A primary renal carcinoma was suspected. ^18^F-FDG PET/CT-scan demonstrated diffuse bone metastasis, hepatic metastasis, lymph node involvement in both the upper and lower regions of the diaphragm, as well as the left corpus luteum (Figure 1). The histological analysis of a retroperitoneal lymph node led to the diagnosis of urothelial carcinoma, with immunohistochemical staining positive for GATA3, CK7, and CK20 (CK20+/), a negative expression of estrogen receptor and HER2 (HER2 0), and a PDL1 level at 15% (Figure 2A–F). The tumor exhibited no loss of mismatch repair (pMMR phenotype). A Fondation One^®^ test on tumor biopsy was requested in November 2023, which concluded an *EGFR* mutation and identified CDK6 amplification, HGF amplification, and CDKN2A/B rearrangement. The results were obtained two months later. Due to the symptomatic disease and the histopathological diagnosis of primary urothelial carcinoma, platinum-based polychemotherapy (methotrexate, vinblastine, adriamycin, and cisplatin) as first-line therapy was started, but no clinical response was observed after three cycles (Figure 1). In January 2024, consistent with the FMI results, the plasma liquid biopsy also revealed a deletion of 24 nucleotides in exon 19 of the *EGFR* gene, specifically *Ala750_Ile759delinsGlyGly*. As reported in lung cancer, we suggested that *EGFR* alteration could be a potential oncogenic driver, so in February 2024, osimertinib (80 mg daily), a third-generation *EGFR* tyrosine kinase inhibitor, was started as a second-line treatment. One month later, a follow-up 18F-FDG PET/CT-scan showed a morpho-metabolic complete response (Figure 1). Note that after one month of treatment, the patient presented hematological toxicity with an isolated prolonged grade 2 thrombopenia despite dose reduction. Heparin-induced thrombocytopenia was excluded, and bone marrow revealed no tumor osteomedullary invasion. In July 2024, she developed liver metastasis, suggesting an acquired resistance to osimertinib (Figure 1). Osimertinib dose was increased without therapeutic efficacy. Since the patient presented a primary resistance to polychemotheray, an acquired resistance to osimertinib after five months of treatment, a positive PDL1 expression, and an analogy of therapeutic management of urothelial carcinoma, pembrolizumab, a check-point inhibitor, was introduced as third-line therapy. However, after the first infusion, the patient succumbed to a pulmonary embolism. Due to her sudden death, no further liquid biopsy could be performed to evaluate the molecular profile to explain acquired resistance to osimertinib.

## 3. Discussion

This case reports an *EGFR* mutation in a young woman diagnosed with metastatic urothelial carcinoma and treated by osimertinib, a third-generation EGFR TKI with a 5-month duration of response despite no efficacy of platinum-based chemotherapy. This rare presentation highlighted several challenges in terms of diagnostic and therapeutic management. Firstly, urothelial carcinoma diagnoses are uncommon in young women without risk factors [1,2,3,4]. Secondly, the association between *EGFR* mutation and urothelial carcinoma is also uncommon. Moreover, in urothelial carcinoma, whether *EGFR* mutation should be considered an oncogenic driver as well as the therapeutic management in the case of uncommon *EGFR* mutation associated with comutations remains also unclear. One similar case of a young woman diagnosed with an *EGFR*-mutated metastatic urothelial carcinoma with a 6-month duration of response to osimertinib, was reported in the current literature [5].

The *EGFR* gene, located on chromosome 7, encodes the first tyrosine kinase receptor described in medical history, which is the central member of the HER family. Its highly conserved structure consists of 1210 amino acids, divided into an extracellular N-terminal domain responsible for ligand binding, a transmembrane domain, a juxtamembrane segment, and an intracellular kinase domain composed of the N-lobe and C-lobe [6]. The receptor interacts with a variety of ligands, including EGF, transforming growth factor alpha (TGF-α), amphiregulin, betacellulin, epigen, epiregulin, and heparin-binding EGF-like growth factor, each of which binds with different affinities and activates distinct downstream signaling pathways [7]. Among these, the Ras/MAPK, STAT3, and PI3K pathways lead to cell activation, differentiation, and proliferation. Dysregulation of *EGFR* signaling often results in tumorigenesis [8]. One of the key mechanisms by which *EGFR* contributes to oncogenesis is through gene amplification, which leads to EGFR overexpression detectable by immunohistochemistry (IHC) [9].

In non-small cell lung cancer (NSCLC), *EGFR* alterations act as oncogenic drivers, revolutionizing patient outcomes with the use of tyrosine kinase inhibitors (TKIs) targeting *EGFR* [10]. L858R EGFR mutation and the deletion in exon 19 (del19) of the *EGFR* gene are the most common and are considered as classical *EGFR* alterations in NSCLC. The typical pattern of del19 alteration is a frame-shift deletion involving five codons (15 nucleotides), corresponding to the sequence E746 to A750 (E746_A750del), which shortens the sequence between the β3 domain and the αC helix, keeping the receptor in its active conformation [11]. In contrast, our case presents an uncommon del19 mutation directly affecting the C-helix. Osimertinib, an oral irreversible third-generation EGFR TKI, is the standard of treatment in L8585 and classical del19 *EGFR*-mutated NSCLC in first-line metastatic setting on the basis of the results of FLAURA trial [10], as well as in second line after first or second generation EGFR TKI, in EGFR T790M positive NSCLC [12]. The substitution of an amino acid threonine to methionine, at the “gatekeeper” 790 residue in exon20 of EGFR, was identified as an acquired resistance to first- and second-generation inhibitors in approximately 50% of the cases through the alteration of inhibitor specificity in the ATP-binding pocket of the protein [13]. Data regarding the therapeutic response to *EGFR* TKIs in the context of uncommon del19 mutations are still debated in NSCLC [14,15,16], as responses may vary based on the specific nature of the del19 alteration, which could modulate the receptor’s affinity for *EGFR* TKIs. Wang et al. conducted a pooled analysis of 196 patients with uncommon *EGFR* mutations in a NSCLC population treated with either afatinib (N = 125) or osimertinib (N = 71) [17]. After propensity score matching, the overall objective response was marginally higher in the afatinib group (60.6% vs. 50.3%, *p* = 0.610). Afatinib conferred a progression-free survival (PFS) benefit (11 vs. 7 months, *p* = 0.039). The authors concluded that both afatinib and osimertinib exhibit favorable tumor responses in NSCLC patients harboring uncommon EGFR mutations. The phase II KCSG-LU15-09 trial [18] reported an 83% response rate at six weeks, with a median duration of response of 11.2 months (95% CI, 7.7–14.7 months) in 37 NSCLC patients with *EGFR* mutations other than exon 19 deletion, L858R, T790M, or exon 20 insertion, treated with osimertinib. The median PFS was 8.2 months (95% CI, 5.9–10.5 months). In contrast to classical *EGFR* alterations (del19 or L858R), survival outcomes remain less favorable regardless of the EGFR TKI used. In the future, novel therapies are needed in case of uncommon EGFR alterations.

Additionally, co-occurring genetic alterations may influence the response to targeted therapies, with negative prognostic significance associated with TP53 [19] or PIK3CA co-mutations [20]. However, our case exhibited a prolonged response to osimertinib despite harboring an uncommon del19 mutation and concomitant alterations in CDK6 amplification, HGF amplification, and CDKN2A/B rearrangement. Literature data distinguished primary resistance [21] to osimertinib, defined as no clinical benefit and/or radiological progressive disease within six months since the beginning of EGFR-inhibitor and acquired resistance. In case of primary resistance, uncommon *EGFR* alterations and concomitant genetic alterations, mainly co-alterations of cell cycle genes such as CDK4/6, are described. The presence of co-mutations of CDK6, HGF and CDKN2A/B in this case could explain the progressive disease after 5 months. Preclinical data suggested the rationale for using a CDK4/6 inhibitor, to treat *EGFR*-mutated NSCLC patients progressed to osimertinib either as a single treatment or combined with osimertinib. Currently, no guidelines recommend this combination in patients with *EGFR*-mutated carcinoma. Moreover, the tolerance profile is not known. Acquired resistance mechanisms to osimertinib are classified according to biological criteria: on-target resistance, off-target resistance, or histological transformation. Off-target resistance mechanisms are led by the activation of an alternative molecular pathway able to fuel cancer cell survival and proliferation despite EGFR inhibition, such as HGF/MET axis activation and cell cycle aberrations, including CDK6 amplification or CDKN2A/B rearrangement [22], as reported in this case. In this context, MET inhibitors or CDK4/6 inhibitors could be potential therapeutic options. The TATTON trial reported an objective response rate of 33% with the combination of osimertinib and savolitinib (a MET inhibitor) in 69 patients with *EGFR*-mutated, *MET*-amplified NSCLC previously treated with a third-generation EGFR TKI. The median PFS was 5.5 months, with an overall survival rate of 62% at 12 months [23]. The phase III MARIPOSA2 trial confirmed the clinical benefit of amivantamab, a bispecific anti-EGFR/MET antibody, in combination with chemotherapy after disease progression on osimertinib in an *EGFR*-mutated NSCLC population [24]. MET-targeting therapies, thus, represent a promising therapeutic approach in *EGFR*-mutated NSCLC. Preclinical data have shown that targeting CDK4/6 in combination with osimertinib synergistically enhances cell growth inhibition [25]. Jager et al. reported a patient diagnosed with metastatic NSCLC harboring a common EGFR mutation, a PIK3CA mutation, and CDK4 amplification, who benefited from combined osimertinib and palbociclib treatment after disease progression on osimertinib alone [26]. Currently, no guidelines recommend the combination of CDK4/6 inhibitor and osimertinib. In the future, bispecific antibody or a combination of targeted therapies could be the therapeutic options in EGFR-comutated lung cancer patients.

Furthermore, checkpoint inhibitors targeting PD-1/PD-L1 are not recommended in metastatic NSCLC patients harboring classical *EGFR* alterations. The role of immunotherapy in uncommon *EGFR* alterations remains unclear. Miyawaki et al. reported that uncommon *EGFR* mutations were significantly associated with a PD-L1 tumor proportion score ≥50% compared to classical *EGFR* alterations. In this cohort, the objective response rate to pembrolizumab was 60% in patients with uncommon *EGFR* mutations and 75% in those with both uncommon *EGFR* mutations and PD-L1 expression ≥50% [27]. A similar result was reported in a patient with an *EGFR*-mutated, PD-L1-positive NSCLC refractory to TKI therapy, who responded to pembrolizumab [28].

In our case, due to the urothelial origin of the carcinoma, the lack of efficacy of polychemotherapy, the acquired resistance to osimertinib, and a positive PDL1 expression, a checkpoint inhibitor was initiated as third-line therapy. However, the benefit of this treatment could not be assessed due to the patient’s sudden death from pulmonary embolism.

In urothelial bladder cancer (UBC), the prevalence of *EGFR* overexpression ranges from 25% to 50%, with a higher frequency observed in muscle-invasive bladder cancer (MIBC) compared to non-muscle-invasive bladder cancer (non-MIBC) [29,30]. *EGFR* overexpression has been consistently linked to poor prognosis [31,32,33,34] and high recurrence rates following curative therapy [35,36]. Blehm et al. found no mutations in the kinase domain between exons 18 and 21 in 75 UBC specimens [37], while Chaux et al. explored the relationship between *EGFR* overexpression and mutations in 19 cases of UBC, finding 14 cases (74%) with *EGFR* overexpression but no concurrent *EGFR* mutations [38]. More recently, an analysis of 3753 urothelial carcinoma cases identified one case of an *EGFR* del19 mutation and three cases of *EGFR* L858R mutations, all of which affect the tyrosine kinase domain (TKD), with additional cases of exon 20 insertions (27 cases) [39].

In triple-negative breast cancer (TNBC), *EGFR* amplification is present in nearly half of the cases and is associated with a poorer prognosis [40]. Park et al. analyzed *EGFR* alterations in 151 TNBC patients, identifying three cases with L858R mutations in exon 21, one with a V786M mutation in exon 20, and one with a G719A mutation in exon 18 [41]. Notably, 64% (91/151) of the patients had *EGFR* amplification, and 33% (50/151) exhibited a high gene copy number. In hereditary breast cancer, Weber et al. described eleven *EGFR* mutations in 24 specimens, with seven mutations observed in sporadic cases [42]. In a case of metastatic HER2-amplified breast cancer, Jing et al. reported a patient with an *EGFR* del19 mutation who was treated with gefitinib for one year [43]. However, clinical trials assessing the efficacy of *EGFR* TKIs in breast cancer have yet to demonstrate significant benefit, despite the frequent occurrence of *EGFR* overexpression [44].

In serous ovarian carcinoma, Lassus et al. reported no *EGFR* mutations in exons 18, 19, or 21 in a study of 198 samples, despite finding 17% *EGFR* overexpression and 12% *EGFR* amplification (>five copies per cell), both associated with poor outcomes [45]. In a phase III trial evaluating erlotinib (an *EGFR* TKI) after a platinum-based regimen, among 318 patients, only three had *EGFR* mutations, while approximately 40% showed *EGFR* amplification. However, *EGFR* alterations were linked to worse prognosis but not to response to erlotinib [46]. No trials have yet demonstrated the efficacy of *EGFR* TKIs in ovarian cancer [47,48,49].

*EGFR* alterations are also found in gastrointestinal cancers. In bile duct cancers (BDC), *EGFR* pathway dysregulation is common, with nearly half of tumors showing *EGFR* overexpression and a few cases harboring *EGFR* mutations [50]. Gwak et al. identified three cases of *EGFR* del19 (two K745-E749 deletions and one E746-A750 deletion) among 22 cholangiocarcinoma specimens, which were associated with poor prognosis [51]. Leone et al. reported six cases of *EGFR* mutations among 40 cholangiocarcinoma specimens, including L858R, T790M, and exon 19 mutations [52]. A phase III trial evaluating erlotinib with gemcitabine and oxaliplatin in metastatic BDC was negative, with molecular subgroup analysis showing only two patients with *EGFR* mutations (T790M) treated with chemotherapy alone [53]. In metastatic gallbladder cancer, Soni et al. reported a case with both *EGFR* T790M and TP53 mutations that responded dramatically to erlotinib combined with chemotherapy, achieving a 90% reduction in tumor size and a median PFS of 11 months [54].

In pancreatic adenocarcinoma (PA), *EGFR* amplification is observed in approximately half of cases, while *EGFR* mutations are rare [55]. Ma et al. reported a case of metastatic PA with *EGFR* del19 (p.E746_s752), treated with furmonertinib (a third-generation *EGFR* TKI) as the fifth-line therapy, achieving a median PFS of 4.7 months [56]. Another case with co-occurring KRAS G12V and *EGFR* L750R mutations responded to erlotinib and gemcitabine with seven months of disease control [57].

In metastatic cancers of unknown primary, Mitani et al. described a case with an *EGFR* L858R mutation who achieved an overall survival of 2 years and 9 months after treatment with erlotinib [58].

*EGFR* alterations were first described in central nervous system tumors, particularly glioblastoma (GBM). Approximately 40% of GBM cases show *EGFR* amplification, and 60% exhibit *EGFR* overexpression, both associated with unfavorable outcomes [59,60]. *EGFR* mutations are found in approximately 40% of GBM, primarily in the extracellular domain, most notably as the *EGFRvIII* mutation, affecting exons 1 to 8 [61]. However, no kinase domain mutations have been consistently reported [62]. A recent case report described a GBM patient with an *EGFR* L858R mutation who was treated with almonertinib, achieving 12 months of PFS [63]. In contrast, Boongird et al. reported a GBM case with both *EGFR* T790M and exon 20 insertion mutations that did not respond to osimertinib [64]. To date, *EGFR* TKI treatment has not improved outcomes in GBM, particularly in cases with *EGFR* alterations [65,66,67].

In head and neck squamous cell carcinoma (HNSCC), *EGFR* alterations, including overexpression and mutations, are common and associated with poor prognosis [68]. A meta-analysis of 4,122 patients found that 2.8% harbored *EGFR* mutations in the kinase domain, including 22% with *del19* [69]. Current therapies for HNSCC focus on *EGFR* monoclonal antibodies due to the limited efficacy of TKIs [70,71,72,73].

## 4. Conclusions

This rare case demonstrates the efficacy of osimertinib in a young patient with an *EGFR* del19-mutated metastatic urothelial carcinoma. It underscores the importance of molecular analysis in young, non-smoking patients with cancer, especially when no obvious risk factors are present. The response to *EGFR* TKIs is universally linked to the presence of *EGFR* mutations in the tyrosine kinase domain (TKD), irrespective of the cancer’s primary histology. This case also highlights that *EGFR* amplification and mutation are not necessarily linked as a continuum, and the presence of mutations in the TKD is more predictive of treatment response.

## Data Availability

The original contributions presented in the study are included in the article, further inquiries can be directed to the corresponding authors.
